# Prognostic Role of Elevated Myeloperoxidase in Patients with Acute Coronary Syndrome: A Systemic Review and Meta-Analysis

**DOI:** 10.1155/2019/2872607

**Published:** 2019-06-25

**Authors:** Andrew R. Kolodziej, Mohamed Abo-Aly, Eman Elsawalhy, Charles Campbell, Khaled M. Ziada, Ahmed Abdel-Latif

**Affiliations:** Gill Heart Institute and Division of Cardiovascular Medicine, University of Kentucky and the Lexington VA Medical Center, Lexington, KY, USA

## Abstract

**Background:**

Myocardial inflammation following acute ischemic injury has been linked to poor cardiac remodeling and heart failure. Many studies have linked myeloperoxidase (MPO), a neutrophil and inflammatory marker, to cardiac inflammation in the setting of acute coronary syndrome (ACS). However, the prognostic role of MPO for adverse clinical outcomes in ACS patients has not been well established.

**Methods:**

MEDLINE and Cochrane databases were searched for studies from 1975 to March 2018 that investigated the prognostic value of serum MPO in ACS patients. Studies which have dichotomized patients into a high MPO group and a low MPO group reported clinical outcomes accordingly and followed up patients for at least 30 days to be eligible for enrollment. Data were analyzed using random-effects model. Sensitivity analyses were conducted for quality control.

**Results:**

Our meta-analysis included 13 studies with 9090 subjects and a median follow-up of 11.4 months. High MPO level significantly predicted mortality (odds ratio (OR) 2.03; 95% confidence interval (CI): 1.40-2.94; *P* < 0.001), whereas it was not significantly predictive of major adverse cardiac events and recurrent myocardial infarction (MI) (OR 1.28; CI: 0.92-1.77, *P* = 0.14 and OR 1.23; CI: 0.96-1.58, *P* = 0.101, respectively). Hypertension, diabetes mellitus, and age did not affect the prognostic value of MPO for clinical outcomes, whereas female gender and smoking status have a strong influence on the prognostic value of MPO in terms of mortality and recurrent MI (metaregression coefficient -8.616: 95% CI -14.59 to -2.633, *P* = 0.0048 and 4.88: 95% CI 0.756 to 9.0133, *P* = 0.0204, respectively).

**Conclusions:**

Our meta-analysis suggests that high MPO levels are associated with the risk of mortality and that MPO can be incorporated in risk stratification models that guide therapy of high-risk ACS patients.

## 1. Introduction

Cardiovascular disease is the leading cause of death worldwide [1]. Acute coronary syndrome (ACS) has the worst prognosis among cardiovascular diseases with significant impact on morbidity and mortality. However, ACS patients are a heterogonous population with variable pathologies and clinical outcomes. Methods for risk stratification that incorporate biological variables such as heightened inflammation after cardiac injury are lacking. While troponin and other cardiac markers have been shown to estimate the degree of initial ischemic insult and long-term clinical events [2], the prognostic value of markers of inflammation is not well established.

Cardiomyocyte damage has been shown to initiate a systemic and local inflammatory response that results in worsening cardiac remodeling and long-term cardiac and clinical adverse events [3, 4]. This response initiates the mobilization, recruitment, and activation of neutrophils and other inflammatory cells. Upon activation, neutrophils degranulate and release inflammatory cytokines such as myeloperoxidase (MPO) which aids in the clearance of dead cells and tissues but has been shown to exert atherogenic and adverse vascular effects [5, 6]. A robust body of well-designed, well-controlled foundational studies conducted in humans and animal models collectively supports the premise that inflammation and circulating inflammatory cells after myocardial infarction are detrimental for cardiac recovery [7, 8]. All these properties make MPO a potential prognostic tool for predicting future adverse clinical outcomes in ACS patients.

Studies that have been conducted to evaluate the prognostic value of MPO in ACS patients showed discrepant results [9, 10] and included a heterogeneous patient population, and their sample size was insufficient to provide solid conclusions. Therefore, we conducted this protocol-driven systematic review and meta-analysis to explore the prognostic value of MPO in ACS patients. We focused on studies that included ACS patients and stratified patients' outcomes based on the plasma MPO levels.

## 2. Materials and Methods

We conducted this protocol-driven systematic review and meta-analysis according to the Preferred Reporting Items for Systematic Reviews and Meta-Analyses (PRISMA) [11]. We sought to compare the 30-day prognosis of ACS patients with high vs. low MPO levels. MEDLINE, Scopus, and Cochrane databases were searched from inception of myeloperoxidase until March 2018. Further details about the search strategy and terms are shown in the Supplemental Table 1. The references of relevant papers were also screened for potential eligible studies. The abstracts of the American Heart Association, American College of Cardiology, and European Society of Cardiology were screened over the last 2 years for eligible studies.

To be eligible for inclusion in our analysis, studies had to meet the following criteria: (1) patients are divided according to a cutoff value of serum MPO into “high” and “low,” (2) patients were followed up for at least 30 days, and (3) absolute numbers of clinical outcome events were reported. Exclusion criteria were (1) irretrievable data, (2) review articles and editorials, and (3) studies including less than 50% subjects with an index diagnosis of ACS. ACS was defined as either ST segment elevation myocardial infraction (STEMI), non-STEMI, or unstable angina. STEMI is defined according to previously published criteria [12, 13] or the WHO criteria [14]. Non-STEMI is defined as at least 10-15 minutes of chest pain at rest and elevated biomarkers of myonecrosis, ST-segment deviation, or T-wave abnormalities. Unstable angina was defined as a typical chest pain at rest with new ST segment changes and peak cardiac troponin I levels within the normal range. Prespecified outcomes of our analyses were mortality, major adverse cardiac events (MACE), and recurrent myocardial infarction. Because of the variability of the definition of the composite of MACE, we included only studies that specifically reported MACE or used a traditional definition of its components.

### 2.1. Data Extraction and Critical Appraisal

Two reviewers (A.A-L and M.A) independently screened the full text of the retrieved studies and used a standardized form to extract the data from each study. For each outcome, absolute event numbers were included and results are expressed as a ratio of total participants with complete follow-up. Patients were divided into 2 groups, above and below the median level of MPO. Regarding reports that investigated the same subjects at different follow-up time points, we extracted data pertaining to outcomes from the longest follow-up report. We used the Newcastle-Ottawa quality assessment scale (NOS) to assess the quality of included studies [15].

### 2.2. Statistical Analyses

The prespecified outcomes of our analyses were mortality, major adverse cardiac events (MACE), and recurrent myocardial infarction. Summary estimates were calculated as odds ratios (OR) with 95% confidence intervals (CI) using the random-effects model based on DerSimonian and Laird's meta-analytic statistical method [16]. Considering that the heterogeneity of the included studies might influence the prognostic effects, we prespecified the use of the random-effects model to assess effect sizes. The *I* ^2^ index was used to summarize the proportion of the total variability in the estimate. The *I* ^2^ statistic is derived from the *Q* statistic and describes the percentage of total variation across studies that is due to heterogeneity; values of 25%, 50%, and 75% correspond to low, moderate, and high heterogeneity, respectively [17] [18]. Sensitivity analyses were performed using the one-study-removed and the cumulative analysis methods in order to assess the influence of each study on the overall pooled results of the meta-analysis. We used Egger's test and visual inspection of Funnel plots to assess for publication bias [19].

### 2.3. Metaregression Analysis

Using log-transformed OR as dependent variable, metaregression analyses were performed to determine whether the prognostic value of MPO was modulated by prespecified study-level factors including age and percentage of female gender, patients with index diagnosis ACS, smoker, diabetes mellitus, and hypertension among study populations. Metaregression was performed with unrestricted maximum-likelihood method (inverse variance-weighted regression) on the event rate log-transformed before being used as independent variables in linear metaregression analyses [20]. The statistical level of significance was 2-tailed *P* < 0.05. All statistical analyses were performed using Comprehensive Meta-Analysis version 3.0 software (Biostat Inc., New Jersey, USA).

## 3. Results

**Figure 1:**
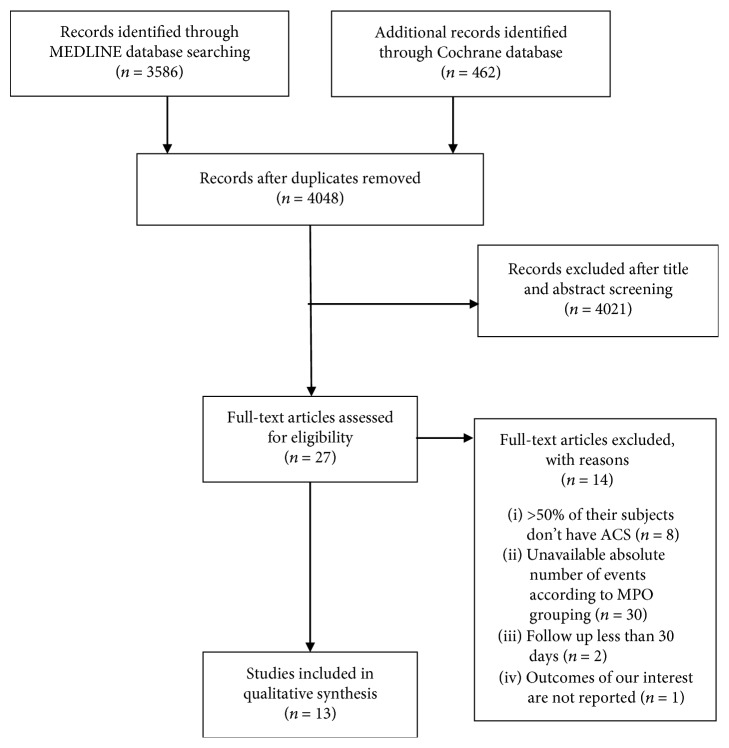
Flow chart of search strategy.

The final analysis included 13 studies that enrolled 9090 subjects with a median follow-up of 11.4 months. The selection process is summarized in Figure 1. Interreviewer agreement on study eligibility was 100%. The baseline characteristics of the included patients are shown in Table 1. Overall, patients with high MPO had similar baseline characteristics compared to those with low MPO. The different definitions of MACE in the included studies in the meta-analysis are shown in Supplemental Table 2. The quality assessment of each included study is shown in Supplemental Table 3.

**Figure 2:**
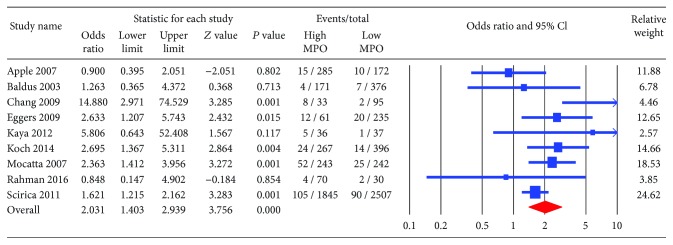
Forest plot for all-cause mortality. High myeloperoxidase level was associated with significantly higher risk of mortality (odds ratio 2.03; 95% confidence interval (CI): 1.403-2.939; *P* < 0.001).

**Figure 3:**
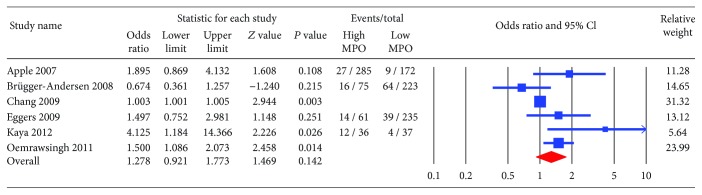
Forest plot for major adverse cardiac events (MACE). High myeloperoxidase showed a trend towards higher risk of MACE (odds ratio 1.27; CI: 0.92-1.77, *P* = 0.14).

**Figure 4:**
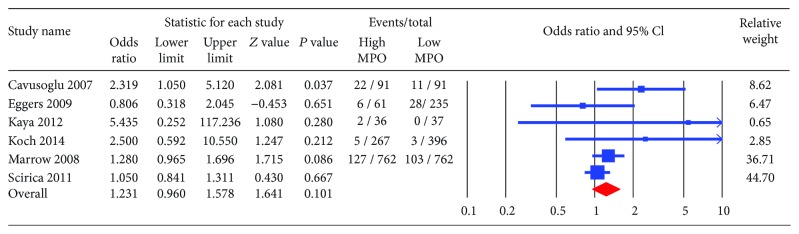
Forest plot for recurrent myocardial infraction (MI). High myeloperoxidase showed a trend towards higher risk of recurrent MI (odds ratio 1.23; CI: 0.96-1.57, *P* = 0.101).

The primary endpoint, all-cause mortality, was significantly higher in patients with high MPO compared to those with low MPO (OR 2.03; CI: 1.40-2.94, *P* < 0.001) (Figure 2). The incidence of MACE and recurrent myocardial infraction trended higher among patients with high MPO (OR 1.28; CI: 0.92-1.77, *P* = 0.14 and OR 1.23; CI: 0.96-1.58, *P* = 0.101, respectively) (Figures 3 and 4). The heterogeneity in our analyses was moderate based on the *I* ^2^ statistic of 17%, 48%, and 77% for recurrent MI, mortality, and MACE, respectively.

Metaregression analysis of the primary endpoints stratified by baseline characteristics, such as age, prevalence of hypertension, percentage of ACS in the study population, and diabetes mellitus, showed no significant interactions (Supplemental Figures 1-3). However, there was a significant inverse correlation between female gender and the prognostic value of MPO for both mortality (correlation coefficient -4.23, 95% CI: -7.88 to -0.59, *P* = 0.02) and recurrent MI (correlation coefficient -2.37, 95% CI: -4.69 to -0.03, *P* = 0.047) (Supplemental Figures 1 and 3). On the other hand, smoking showed a significant direct correlation with the OR of recurrent MI; hence, the prognostic value of high MPO on recurrent MI was greater among smokers than nonsmokers (correlation coefficient 5.21, 95% CI: 1.08 to 9.34, *P* = 0.01) (Supplemental Figure 3).

### 3.1. Sensitivity Analyses

Sensitivity analyses using the “one-study-removed” method did not show significant changes in the summary odds ratio estimates for any outcome assessed (Supplemental Figure 4). Cumulative meta-analysis showed a relatively stable accumulation of evidence for primary endpoints assessed (Supplemental Figure 5). We also stratified the studies based on sample collection method. The results were inconclusive for the sample collection tube because there was a significant imbalance with higher number of studies that utilized EDTA collection tube compared to those using heparin or citrate collection tubes, thus precluding a definitive conclusion regarding the impact of sample collection method on the prognostic value of MPO in our analysis.

We also stratified the studies based on sample collection timing. There was heterogeneity in the sample collection time in relation to the onset of chest pain as detailed in Table 2. There was no correlation between the timing of blood collection and the prognostic value of MPO in mortality (-0.00, 95% CI: -0.02 to 0.02, *P* = 0.99), MACE (-0.02, 95% CI: -0.15 to 0.11, *P* = 0.78), or recurrent MI (-0.00, 95% CI: -0.03 to 0.03, *P* = 0.99).

### 3.2. Publication Bias

No clear evidence of publication bias was observed on visual inspection of the Funnel plots (Supplemental Figures 6-8). Our Egger's regression test did not show significant risk of publication bias (*P* = 0.39 for all-cause mortality, 0.06 for MACE, and 0.2 for recurrent MI).

## 4. Discussion

Risk stratification for patients with ACS is an evolving field, and the prognostic role of inflammatory markers such as MPO has not been fully investigated. In this comprehensive systematic review and meta-analysis, we confirm the strong correlation between elevated plasma MPO levels and cardiac outcomes, including mortality, among patients with acute coronary syndrome. More importantly, our results were consistent across multiple study designs and patient characteristics. These results support a potential role for MPO as part of multimarker risk stratification model to guide future individualized therapies to the highest risk population. Future prospective studies examining the prognostic value of MPO in comparison of other biomarkers of inflammation such as high-sensitivity C-reactive protein (hs-CRP) are warranted.

Myocardial injury triggers a series of signaling events to communicate with the bone marrow and peripheral blood cells (PBCs) through processes that are just now being elucidated. After myocardial infarction, circulating inflammatory cells such as neutrophils are a poor prognostic indicator, in part because of their contribution to infarct expansion and impaired cardiac remodeling, thereby promoting the progression to adverse remodeling and heart failure [21, 22]. Indeed, this initial injury response may actually confer long-term harm because reduction in the initial recruitment of inflammatory cells can reduce infarct size and prevent cardiac remodeling following cardiac injury [23]. In addition to effects on the myocardium, circulating inflammatory cells following ACS accelerate experimental atherosclerosis in animal models thus initiating a vicious cycle; thus, this type of cycle may contribute to recurrent coronary events in humans [24]. Therefore, identifying markers of inflammation and inflammatory cell activity can help risk stratify ACS patients and guide future therapies. MPO is a product of inflammatory neutrophils during their degranulation and can aid in the process of clearing dead cells. However, MPO has been linked to atherosclerosis and recurrent coronary events. MPO enhances LDL cholesterol oxidation, hence destabilizes coronary atherosclerotic plaque [25]. Additionally, MPO limits endothelial-derived nitric oxide bioavailability which impairs coronary vessel dilatation and worsens cardiac ischemia [6].

Myeloperoxidase as a prognostic marker in ACS patients has generated conflicting results in clinical studies. The majority of clinical data has confirmed the prognostic value of MPO in predicting mortality [9, 26–29], and our analysis confirmed this correlation to be highly significant. However, although there was strong correlation between MPO levels and other clinical events such as MACE and recurrent myocardial infarction, this association did not reach statistical significance in individual trials or our analysis [9, 30, 31]. There are multiple factors that can explain the lack of this correlation. Some of the studies were underpowered to reach a valid conclusion especially in individual endpoints [10, 30, 32]. Other studies enrolled a heterogeneous population of patients with chest pain (mixture of ACS and non-ACS). Indeed, it has been shown that MPO levels correlate with the severity of ACS pathology [33]. In accordance with these findings, we found that the prognostic value of MPO was the highest among studies with high proportion of AMI patients [10, 27] compared to those with higher percentage of unstable angina subjects [29, 34]. Additionally, timing of sample collection could have played a role in the variable results since MPO level was significantly higher immediately after STEMI [26, 35]. Although studies adopted different MPO cutoff values, our analysis was primarily focused on the prognostic value of MPO rather than its absolute value since the included studies used different MPO assays. Therefore, despite the fact that MPO cutoff value was not the same, stratifying patients based on a certain MPO cutoff provided valuable prognostic information in patients with acute coronary syndrome.

We performed additional sensitivity analyses attempting to unify the included studies based on methodology and sample collection. When we focused our analyses on studies that reported using similar methodology, we observed consistent prognostic value of MPO for all endpoints examined. Similarly, we did not see significant interaction between most of the baseline characteristics or time of sample collection and the prognostic value of plasma MPO. Additionally, the predictive value of MPO for all-cause mortality and MACE was consistent in the “one-study-removed” and cumulative analyses suggesting the generalizability of our findings.

There are limitations to our analysis inherent in conducting a meta-analysis using published patient data and the methodological differences among the included studies. Included studies enrolled heterogeneous patient populations and adopted different definition of clinical outcomes which could have influenced the results of the pooled analyses. While we attempted to address this limitation by using comprehensive sensitivity and metaregression analyses, we could have failed to include other clinical parameters that were not reported in the published manuscripts. Furthermore, the sample withdrawal timing was different across the included studies which could have influenced the results; however, there was no significant correlation between the time of sample withdrawal and the prognostic value of MPO for any of the outcomes. Finally, statin therapy, which is known to downregulate MPO expression [36], was not reported in most of the included studies, and therefore, we could not conduct sensitivity analysis based on the proportion of patients receiving statin.

This meta-analysis attempted to focus on a homogeneous population of studies with high percentage of ACS patients, thus addressing some of the variability in the literature. Our results have significant implications in clinical practice. Integrating MPO in risk stratification models could have an additional value in identifying patients at higher risk of developing heart failure, recurrent ischemia, and clinical events specially mortality. The predictive value of MPO is more specific in patients with STEMI and high-risk NSTEMI where the damage is higher and more inflammatory cells are more activated [28, 37].

## 5. Conclusions

MPO is a powerful prognostic marker for clinical outcomes in patients with acute coronary syndrome. Our results advocate for more comprehensive risk assessment tools that incorporate MPO to more personalized medical and invasive management for patients with ACS. Further studies examining management strategies based on peak MPO level are needed to assess the clinical utility of this novel biomarker.

## Figures and Tables

**Table 1:** Patients' characteristics of the studies included in the meta-analysis.

	STEMI (%)	NSTEMI (%)	UAP (%)	MPO cutoff value	Sample size	Age	Female (%)	Smoking (%)	Diabetes (%)	Hypertension (%)
Apple et al.^∗^ [31]	≥50% of patients have cTnI ≥ 0.09		NA	≤125.6 mcg/L	172	57 ± 16	43	NR	24.9	57.9
				>125.6 mcg/L	285					

Baldus et al. [34]	0	0	100	<350 *μ*g/L	376	61.4 ± 10.5	28.7	42.5	8.2	35.4
				≥350 *μ*g/L	171	62.5 ± 10.4	31	40	12.5	36.9

Brugger-Andersen et al. [30]	AMI 100		0	≤26.8 mcg/L	142	64 ± 13	20.8	38.9	10.4	24.4
				>26.8 mcg/L	141					

Cavusoglu et al. [38]	12	43	45	≤20.34 ng/mL	91	65 ± 9.3	0.0	32	59	84
				>20.34 ng/mL	91	64.7 ± 10.8	0.0	43	34	83

Chang et al. [28]	AMI 53.9		NA	<1150 ng/mL	95	59.9 ± 12.8	10.55	34.7	33.7	60
				≥1150 ng/mL	33	64.3 ± 12.1	15.1	39.4	51.5	57.6

Eggers et al. [9]	AMI 36.6		21.8	≤208.1 pmol/L	235	66 (55, 76)	33.9	17.2	16.2	37.3
				>208.1 pmol/L	61					

Kaya et al. [10]	100	0	0	≤68 ng/mL	37	56 ± 11	26	61	20	37
				>68 ng/mL	36	57 ± 13	21	66	32	55

Koch et al.^§^ [26]	43	NA	NA	≤306.3 pmol/L	396	63.7 ± 13.0	30.3	32.9	19.2	70.9
				>306.3 pmol/L	267	65 ± 12	31.1	33	23.6	67.8

Morrow et al. [39]	0	35	65	≤884 pg/mL	762	61 (52, 69)	32.1	28.7	25.1	67.2
				>884 pg/mL	762	61 (53, 70)	34	29.5	28.9	63.9

Mocatta et al. [27]	81.1	18.9	0	≤55 ng/mL	242	61.7 ± 11.0	19.9	NA	12.7	NR
				>55 ng/mL	243					

Oemrawsingh^†^ et al. [40]	0	0	100	<350 *μ*g/L	376	62 (54, 69)	20	40	14	42
				≥350 *μ*g/L	171					

Rahman et al. [32]	65	30	5	<285.5 pmol/L	30	NR	20	NR	NR	NR
				≥285.5 pmol/L	70					

Scirica et al. [29]	0	48.3	49.2	≤670 pg/mL	2507	64	35.1	25	32.3	74.6
				>670 pg/mL	1845					

STEMI: ST-elevation myocardial infarction; NSTEMI: non-ST-elevation myocardial infarction; UAP: unstable angina; MPO: myeloperoxidase; CTn1: cardiac troponin I; AMI: acute myocardial infraction; NA: not available. Continuous variables are presented in either median or mean ± SD. Categorical variables are presented in percentages. ^∗^Apple et al. reported that the median cardiac troponin of the whole cohort is 0.09 *μ*g/L. ^†^Oemrawsingh et al. is a longer follow-up report of Baldus et al.'s study subjects. ^§^Reported that ACS-negative patients are 10.8% of the study population.

**Table 2:** Sample collection methods and time of sample collection in relation to onset of chest pain/hospital admission of the included studies.

	Apple et al. [31]	Baldus et al.^∗^ [34]	Brugger-Andersen et al. [30]	Cavusoglu et al. [38]	Chang et al. [28]	Eggers et al. [9]	Kaya et al. [10]	Koch et al. [26]	Mocatta et al. [27]	Morrow et al. [39]	Rahman et al. [32]	Scirica et al. [29]
Sample collection method	Heparin-containing tubes	NR	Citrate-anticoagulated tubes	NR	NR	EDTA-anticoagulated tubes	NR	EDTA-anticoagulated tubes	EDTA-anticoagulated tubes	Citrate-anticoagulated tubes	NR	EDTA-anticoagulated tubes
Time of sample collection	Within 3.1 hours from the onset of symptoms	Within 8.7 hours from the onset of symptoms	Within 4-6 days from the onset of symptoms	≥12 hours after hospital admission	At hospital admission	After 0.8 hour from hospital admission	Within 6 hours of the onset of symptoms	After 26.1 hours from hospital admission	From 24 to 96 hours after hospital admission	NR	NR	NR

^∗^Oemrawsingh et al. is a longer follow-up report of Baldus et al.'s study; the study's subjects received heparin before blood samples were withdrawn.

## Supplementary Materials

Supplementary Materials Supplemental Table 1: (A) MEDLINE search strategy. (B) Scopus search strategy. Supplemental Table 2: the definition of major adverse cardiac events of the included studies in the meta-analysis. Supplemental Table 3: assessment of the quality of the included studies using the Newcastle-Ottawa quality assessment scale. Supplemental Figure 1: metaregression for risk factors of mortality. *X* axis represents the observed effect size of studies. *Y* is the metaregression coefficient. Age (*Y* = −0.12; *P* = 0.32). Female (-8.61; *P* = 0.0048). ACS (*Y* = −0.071; *P* = 0.94). DM (*Y* = 3.522; *P* = 0.32). Hypertension (*Y* = −0.20; *P* = 0.911). Smoking (*Y* = 1.44; *P* = 0.55). Supplemental Figure 2: metaregression for risk factors of major adverse cardiac events. *X* axis represents the observed effect size of studies. *Y* is the metaregression coefficient. Age (*Y* = −0.112; *P* = 0.28). Female (-3.15; *P* = 0.301). ACS (*Y* = −0.22; *P* = 0.897). DM (*Y* = −0.353; *P* = 0.89). Hypertension (*Y* = −0.74; *P* = 0.785). Smoking (*Y* = 2.04; *P* = 0.28). Supplemental Figure 3: metaregression for risk factors of recurrent myocardial infraction. *X* axis represents the observed effect size of studies. *Y* is the metaregression coefficient. Age (*Y* = −0.03; *P* = 0.68). Female (-2.23; *P* = 0.06). ACS (*Y* = −0.11; *P* = 0.89). DM (*Y* = 2.0; *P* = 0.286). Hypertension (*Y* = 1.13; *P* = 0.37). Smoking (*Y* = 4.8; *P* = 0.204). Supplemental Figure 4: forest plot displays sensitivity analysis using the one-study-removed method. High myeloperoxidase is significantly associated with mortality (odds ratio 2.040; 95% confidence interval (CI): 1.405-2.960, *P* = 0.000). High MPO showed a trend for developing major adverse cardiac events (odds ratio 1.421; 95% confidence interval (CI): 1.010-1.999, *P* = 0.044) and recurrent MI (odds ratio 1.241; 95% confidence interval (CI): 0.996-1.545, *P* = 0.054). Supplemental Figure 5: forest plot displays cumulative meta-analysis. High myeloperoxidase is significantly associated with mortality (odds ratio 2.040; 95% confidence interval (CI): 1.405-2.960, *P* = 0.000). High MPO showed a trend for developing major adverse cardiac events (odds ratio 1.421; 95% confidence interval (CI): 1.010-1.999, *P* = 0.044) and recurrent MI (odds ratio 1.241; 95% confidence interval (CI): 0.996-1.545, *P* = 0.054). Supplemental Figure 6: funnel plot of all studies included in the meta-analysis. The standard error (SE) of the log odds ratio of each study was plotted against the odds ratio for mortality. No skewed distribution was observed, suggesting no publication bias. Supplemental Figure 7: funnel plot of all studies included in the meta-analysis. The standard error (SE) of the log odds ratio of each study was plotted against the odds ratio for major adverse cardiac events. No skewed distribution was observed, suggesting no publication bias. Supplemental Figure 8: funnel plot of all studies included in the meta-analysis. The standard error (SE) of the log odds ratio of each study was plotted against the odds ratio for recurrent myocardial infraction. No skewed distribution was observed, suggesting no publication bias.
