# Supplementary material for: Prognostic Role of Elevated Myeloperoxidase in Patients with Acute Coronary Syndrome: A Systemic Review and Meta-Analysis
Source: Mediators Inflamm. 2019 Jun 25;2019:2872607. doi: 10.1155/2019/2872607 (PMC6614978; doi:10.1155/2019/2872607)
Supplement: Supplementary Materials — Supplemental Table 1: (A) MEDLINE search strategy. (B) Scopus search strategy. Supplemental Table 2: the definition of major adverse cardiac events of the included studies in the meta-analysis. Supplemental Table 3: assessment of the quality of the included studies using the Newcastle-Ottawa quality assessment scale. Supplemental Figure 1: metaregression for risk factors of mortality. X axis represents the observed effect size of studies. Y is the metaregression coefficient. Age (Y = −0.12; P = 0.32). Female (-8.61; P = 0.0048). ACS (Y = −0.071; P = 0.94). DM (Y = 3.522; P = 0.32). Hypertension (Y = −0.20; P = 0.911). Smoking (Y = 1.44; P = 0.55). Supplemental Figure 2: metaregression for risk factors of major adverse cardiac events. X axis represents the observed effect size of studies. Y is the metaregression coefficient. Age (Y = −0.112; P = 0.28). Female (-3.15; P = 0.301). ACS (Y = −0.22; P = 0.897). DM (Y = −0.353; P = 0.89). Hypertension (Y = −0.74; P = 0.785). Smoking (Y = 2.04; P = 0.28). Supplemental Figure 3: metaregression for risk factors of recurrent myocardial infraction. X axis represents the observed effect size of studies. Y is the metaregression coefficient. Age (Y = −0.03; P = 0.68). Female (-2.23; P = 0.06). ACS (Y = −0.11; P = 0.89). DM (Y = 2.0; P = 0.286). Hypertension (Y = 1.13; P = 0.37). Smoking (Y = 4.8; P = 0.204). Supplemental Figure 4: forest plot displays sensitivity analysis using the one-study-removed method. High myeloperoxidase is significantly associated with mortality (odds ratio 2.040; 95% confidence interval (CI): 1.405-2.960, P = 0.000). High MPO showed a trend for developing major adverse cardiac events (odds ratio 1.421; 95% confidence interval (CI): 1.010-1.999, P = 0.044) and recurrent MI (odds ratio 1.241; 95% confidence interval (CI): 0.996-1.545, P = 0.054). Supplemental Figure 5: forest plot displays cumulative meta-analysis. High myeloperoxidase is significantly associated with mortality (odds ratio 2.040; 95% confid [file 2872607.f1.pdf]

Supplemental Table 1. A MEDLINE search strategy:

#	Search	Results
#1	"Peroxidase"[Mesh]	134863
#2	“Biomarkers" [Mesh]	800721
#3	"Inflammation Mediators"[Mesh]	361740
#4	#1 OR #2 OR #3	1137062
#5	"Myocardial Infarction” [Mesh]	161335
#6	"Acute Coronary Syndrome" [Mesh]	11907
#7	"Non-ST Elevated Myocardial Infarction" [Mesh]	211
#8	"Angina, Unstable"[Mesh]	10563
#9	#5 OR #6 OR #7 OR #8	175986
#10	"Prognosis"[Mesh]	1368644
#11	"Predictive Value of Tests"[Mesh])	175855
#12	#10 OR #11	1487900
#13	#4 And #9 And #12	3586

Supplemental Table 1.B Scopus Search Strategy

#	Search terms	Results
	(ALL (peroxidase) OR ALL (myeloperoxidase) OR ALL (biomarkers)) AND (ALL (prognosis)) AND (ALL (myocardial AND infarction) OR ALL (acute AND coronary AND syndrome) OR ALL (non-st AND elevated AND myocardial AND infarction) OR ALL (unstable AND angina)) AND NOT (ALL (cancer) OR ALL (carcinoma) OR ALL (aortic AND stenosis) OR ALL (mice) OR ALL (thyroid) OR ALL (pancreas) OR ALL (renal) OR ALL (liver) OR ALL (kidney) OR ALL (leukemia) OR ALL (tumor) OR ALL (immunohistochemistry) OR ALL (uterus) OR (animals) OR (prostate) OR ALL (in AND vitro) OR ALL (infection) OR ALL (lymphoma) OR ALL (epithelial AND cells) OR ALL (lung) OR ALL (gene) OR ALL (genetic) OR ALL (rabbit) OR ALL (rats) OR ALL (bone) OR ALL (mineral) OR (intestinal) OR (intestine) OR ALL (pulmonary) OR ALL (environment) OR ALL (brain) OR ALL (sepsis) OR ALL (psychosis) OR ALL (colon) OR ALL (case AND report) OR (arthritis)) AND (LIMIT-TO (LANGUAGE , "English")) AND (LIMIT-TO (SUBJAREA , "MEDI"))	**904**

Supplemental Table 2. **The definition of Major adverse cardiac events of the included studies in the meta-analysis.**

Study	Major adverse cardiac events (MACE) definition
**Apple**[20]	A composite endpoint of the first recurrent MI, PCI, CABG, or cardiac death.
**Brügger Andersen**[25]	Recurrent TnT-positive ACS or cardiac death.
**Chang** [26]	A composite endpoint of advanced Killip score (defined as Killip classification ≥ 3) upon presentation, re-infarction, repeat PCI or mortality.
**Eggers** [9]	A composite endpoint of mortality and acute MI.
**Kaya** [10]	cardiac death; reinfarction; new hospital admission for angina; heart failure; and revascularization procedures by means of CABG or PCI.
**Oemrawsingh** [23]	A composite endpoint of all-cause mortality and non-fatal MI.

MI, Myocardial infraction; PCI, percutaneous coronary intervention; CABG, coronary artery bypass graft; TnT, Cardiac troponin.
T; ACS, Acute coronary syndrome.

Supplemental Table 3. Assessment the quality of the included studies using the Newcastle-Ottawa quality assessment scale:

Study	Selection				Comparability	Ascertainment of outcome			Total Score
	Representativeness of the exposed cohort	Selection of the non-exposed cohort	Ascertainment of exposure	Demonstration that outcome of interest was not present at start of the study	Comparability of cohorts on the basis of the design or analysis	Independent assessment or record linkage	Follow up long enough for outcomes to occur	Adequacy of follow up of cohort	
Apple [31]	*	*	*	*	*	*	*	*	8
Baldus [34]	*	*	*	*	*	#	*	*	7
Brügger-Andersen [30]	*	*	*	*	**	*	*	*	9
Cavusoglu [38]	*	*	*	*	**	*	*	*	9
Chang [28]	*	*	*	*	*	#	$	*	6

Eggers [9]	*	*	*	*	*	*	*	*	8
Kaya [10]	*	*	*	*	*	*	*	*	8
Koch [26]	*	*	*	*	**	#	*	*	8
Morrow [39]	*	*	*	*	*	*	*	*	8
Mocatta [40]	*	*	*	*	*	*	*	*	8
Oemrawsingh [41]	*	*	*	*	**	*	*	*	9
Rahman [32]	*	*	*	*		#	*	*	6
Scirica [29]	*	*	*	*	*	*	*	*	8

No evidence of independent blind assessment of outcomes or linkage to patient’s record, however we believe that the lack of blinding could have less likely introduced a risk of bias in evaluating our outcomes of interest. $ follow up is 30 days. A maximum two stars can be given for comparability based on the number of risk factors they adjusted for.

Supplemental Figure Legends:

Supplemental Figure 1. Meta-regression for risk factors of Mortality. X axis represents the observed effect size of studies. Y is Meta-regression coefficient. Age (Y= - 0.12; P=0.32). Female (-8.61; P= 0.0048). ACS (Y=-0.071; P=0.94). DM (Y=3.522; P=0.32). Hypertension (Y-0.20; P=0.911). Smoking (Y=1.44; P=0.55).

Supplemental Figure 2. Meta-regression for risk factors of Major Adverse cardiac events. X axis represents the observed effect size of studies. Y is Meta-regression coefficient. Age (Y= - 0.112; P=0.28). Female (-3.15; P= 0.301). ACS (Y=-0.22; P=0.897). DM (Y= -0.353; P=0.89). Hypertension (Y-0.74; P=0.785). Smoking (Y=2.04; P=0.28).

Supplemental Figure 3. Meta-regression for risk factors of Recurrent Myocardial infraction. X axis represents the observed effect size of studies. Y is Meta-regression coefficient. Age (Y= - 0.03; P=0.68). Female (-2.23; P= 0.06). ACS (Y=-0.11; P=0.89). DM (Y= 2.0; P=0.286). Hypertension (Y1.13; P=0.37). Smoking (Y=4.8; P=0.204).

Supplemental Figure 4. Forest Plot displays Sensitivity analysis using the one study removed method. High myeloperoxidase is significantly associated with mortality (odds ratio 2.040; 95% confidence interval [CI]: 1.405-2.960, p=0.000). High MPO showed a trend for developing major adverse cardiac events (odds ratio 1.421; 95% confidence interval [CI]: 1.010-1.999, p=0.044) and recurrent MI (odds ratio 1.241; 95% confidence interval [CI]: 0.996-1.545, p=0.054).

Supplemental Figure 5. Forest Plot displays Cumulative meta-analysis. High myeloperoxidase is significantly associated with mortality (odds ratio 2.040; 95% confidence interval [CI]: 1.405-2.960, p=0.000). High MPO showed a trend for developing major adverse

cardiac events (odds ratio 1.421; 95% confidence interval [CI]: 1.010-1.999, p=0.044) and recurrent MI (odds ratio 1.241; 95% confidence interval [CI]: 0.996-1.545, p=0.054).

Supplemental Figure 6. Funnel plot of all studies included in the meta-analysis. The Standard Error (SE) of the log Odds Ratio of each study was plotted against the Odds Ratio for mortality. No skewed distribution was observed, suggesting no publication bias.

Supplemental Figure 7. Funnel plot of all studies included in the meta-analysis. The Standard Error (SE) of the log Odds Ratio of each study was plotted against the Odds Ratio for major adverse cardiac events. No skewed distribution was observed, suggesting no publication bias.

Supplemental Figure 8. Funnel plot of all studies included in the meta-analysis. The Standard Error (SE) of the log Odds Ratio of each study was plotted against the Odds Ratio for recurrent myocardial infraction. No skewed distribution was observed, suggesting no publication bias.

Mortality

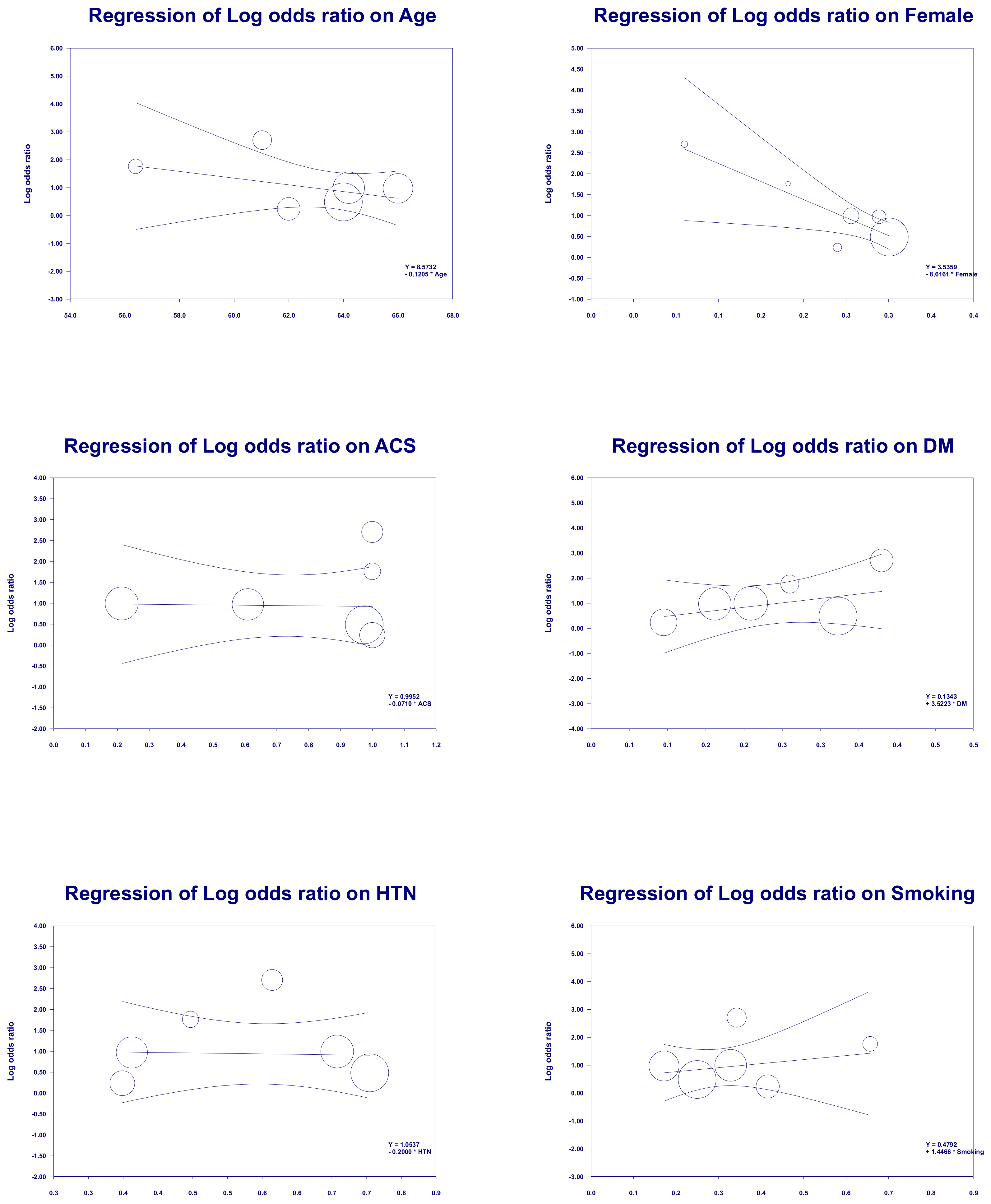

Supplemental Figure 1.

Major adverse cardiac events

Regression of Log odds ratio on Age

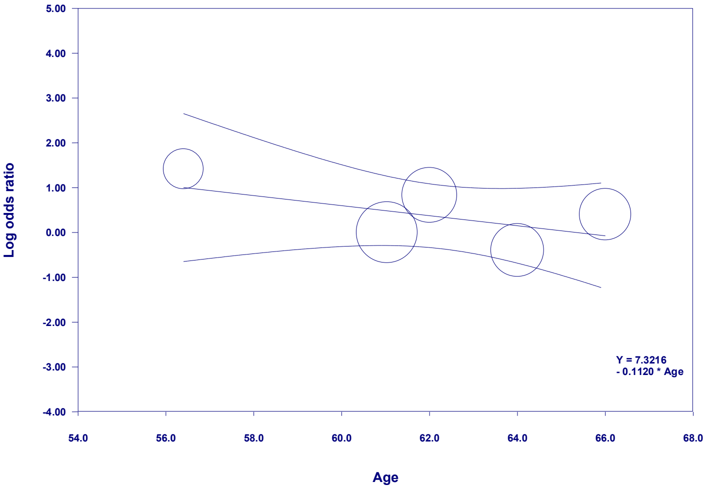

Regression of Log odds ratio on Female

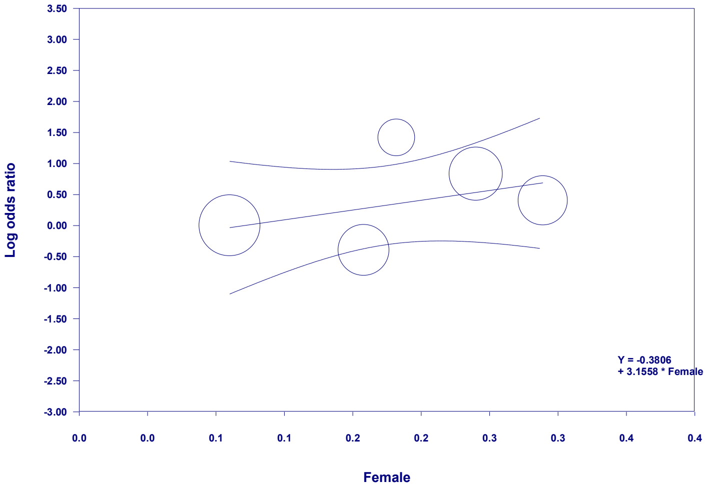

Regression of Log odds ratio on ACS

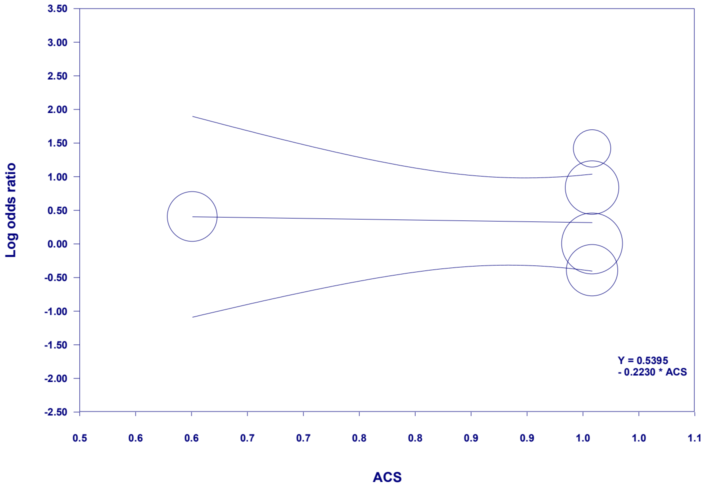

Regression of Log odds ratio on DM

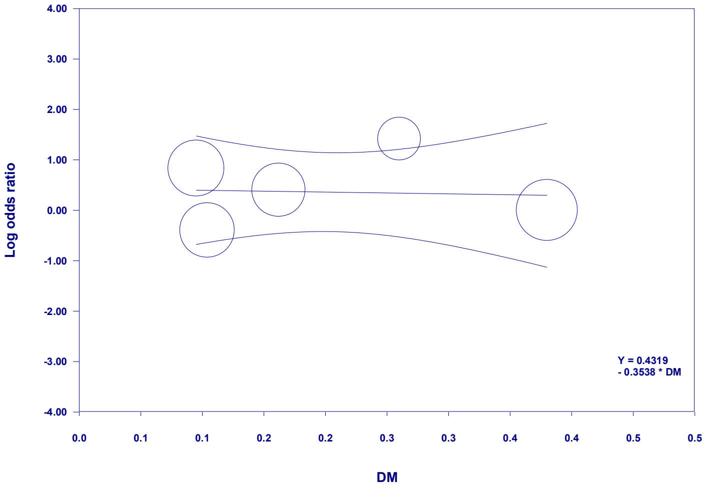

Regression of Log odds ratio on HTN

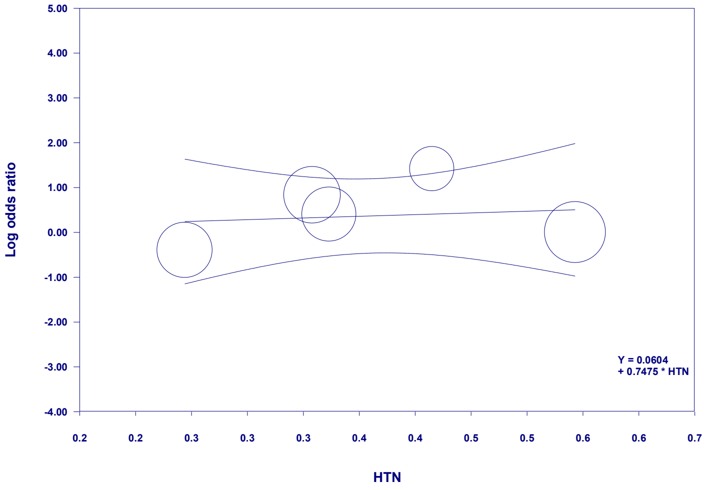

Regression of Log odds ratio on Smoking

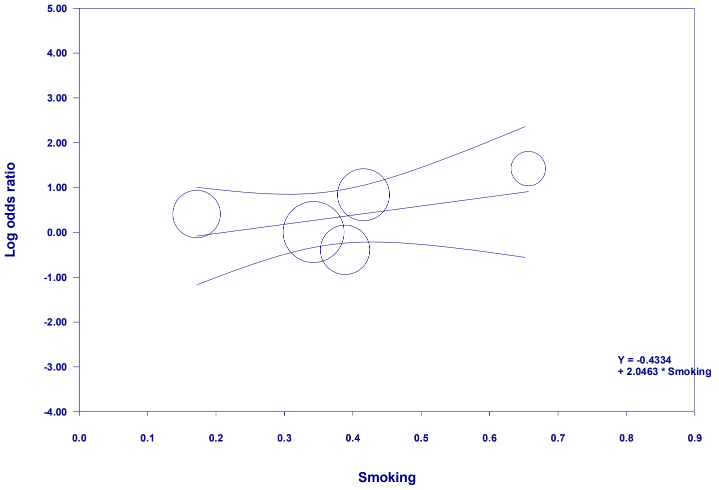

Supplemental Figure 2.

Recurrent myocardial infarction

Regression of Log odds ratio on Age

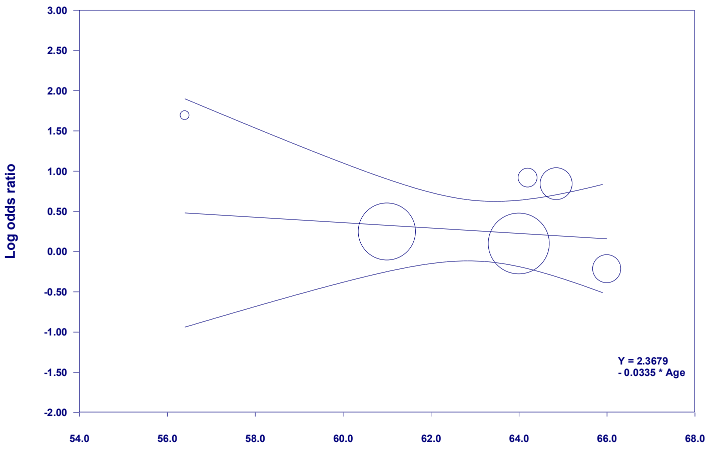

Regression of Log odds ratio on Female

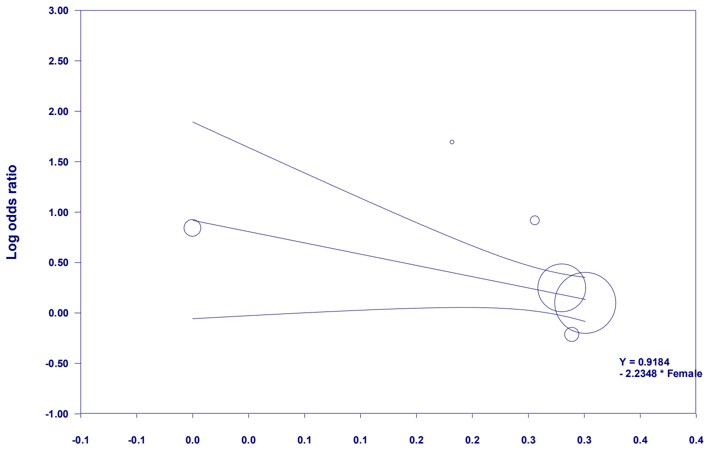

Regression of Log odds ratio on ACS

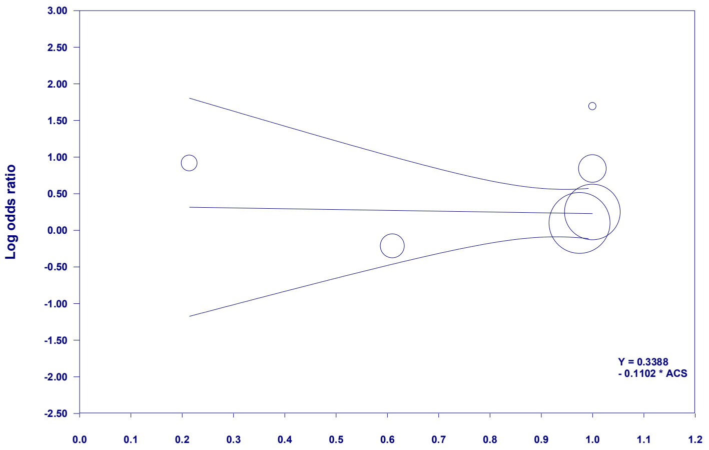

Regression of Log odds ratio on DM

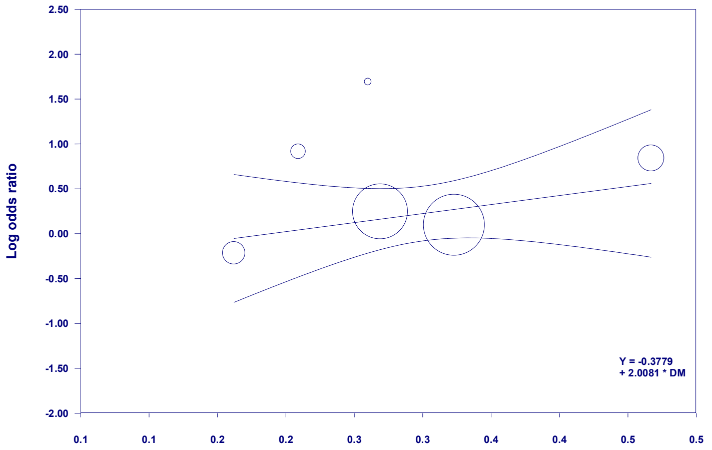

Regression of Log odds ratio on HTN

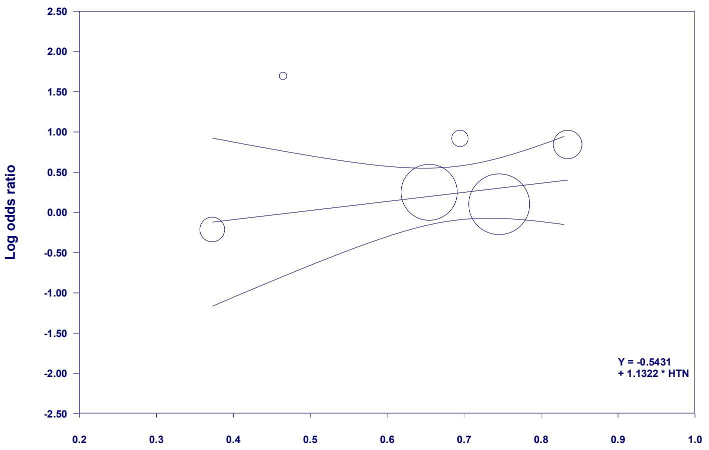

Regression of Log odds ratio on Smoking

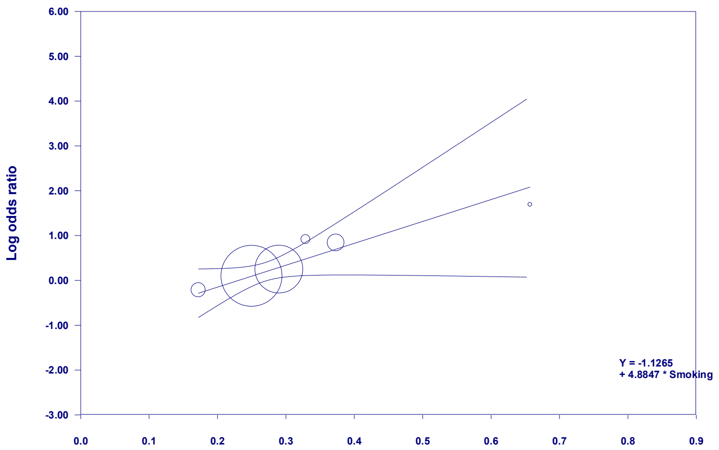

Supplemental Figure 3.

Odds ratio (95% CI) with study removed

A. Mortality

Study name	Point	Lower limit	Upper limit	Z-Value	p-Value
Baldus 2003	2.125	1.423	3.175	3.682	0.000
Chang 2009	1.852	1.400	2.451	4.315	0.000
Eggers 2009	1.976	1.299	3.005	3.185	0.001
Mocatta 2007	1.992	1.269	3.126	2.997	0.003
Kaya 2012	1.984	1.358	2.898	3.543	0.000
Scirica BM 2011	2.209	1.371	3.559	3.257	0.001
Rahman 2016	2.119	1.440	3.117	3.813	0.000
Koch 2014	1.955	1.282	2.983	3.111	0.002
Apple 2007	2.253	1.552	3.270	4.271	0.000
	2.040	1.405	2.960	3.751	0.000

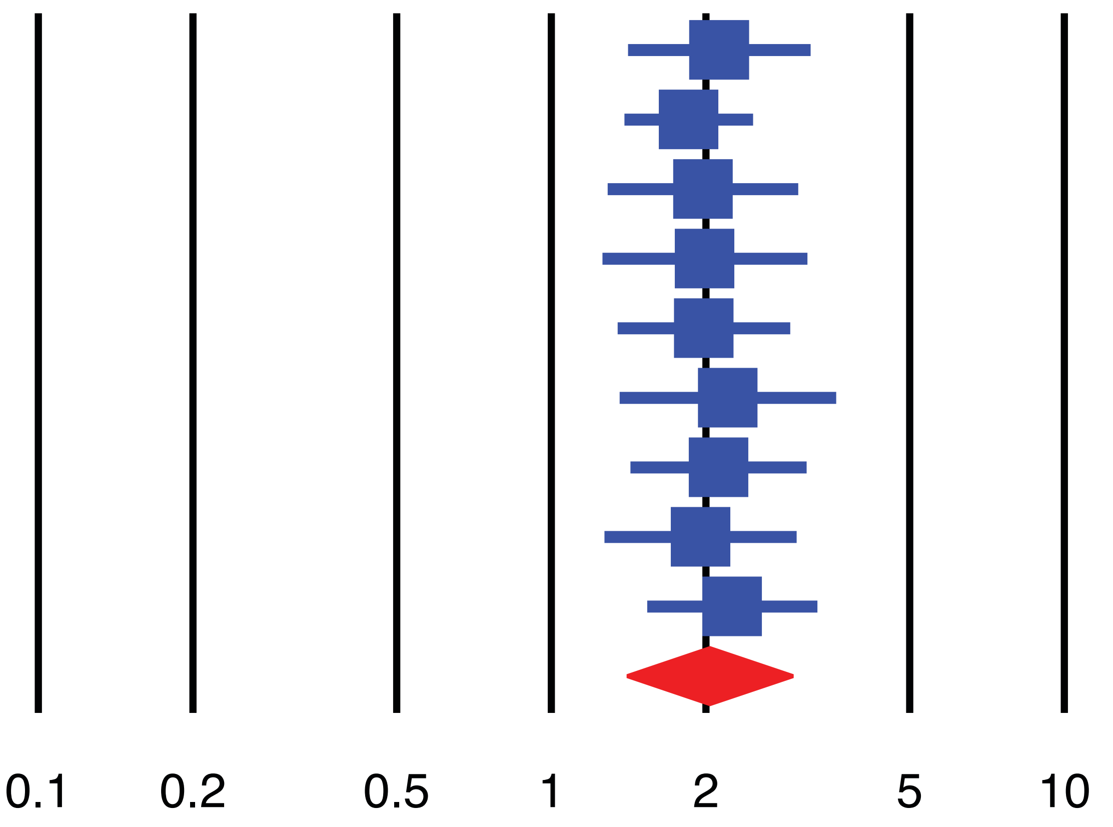

B. MACE

Study name	Point	Lower limit	Upper limit	Z-Value	p-Value
**Chang 2009**	1.581	1.075	2.325	2.330	0.020
**Brügger 2008**	1.605	1.096	2.349	2.432	0.015
**Eggers 2009**	1.418	0.973	2.067	1.815	0.069
**Kaya 2012**	1.326	0.954	1.842	1.680	0.093
**Oemrawsingh 2011**	1.434	0.933	2.204	1.642	0.101
**Apple 2007**	1.373	0.956	1.971	1.718	0.086
	1.421	1.010	1.999	2.014	0.044

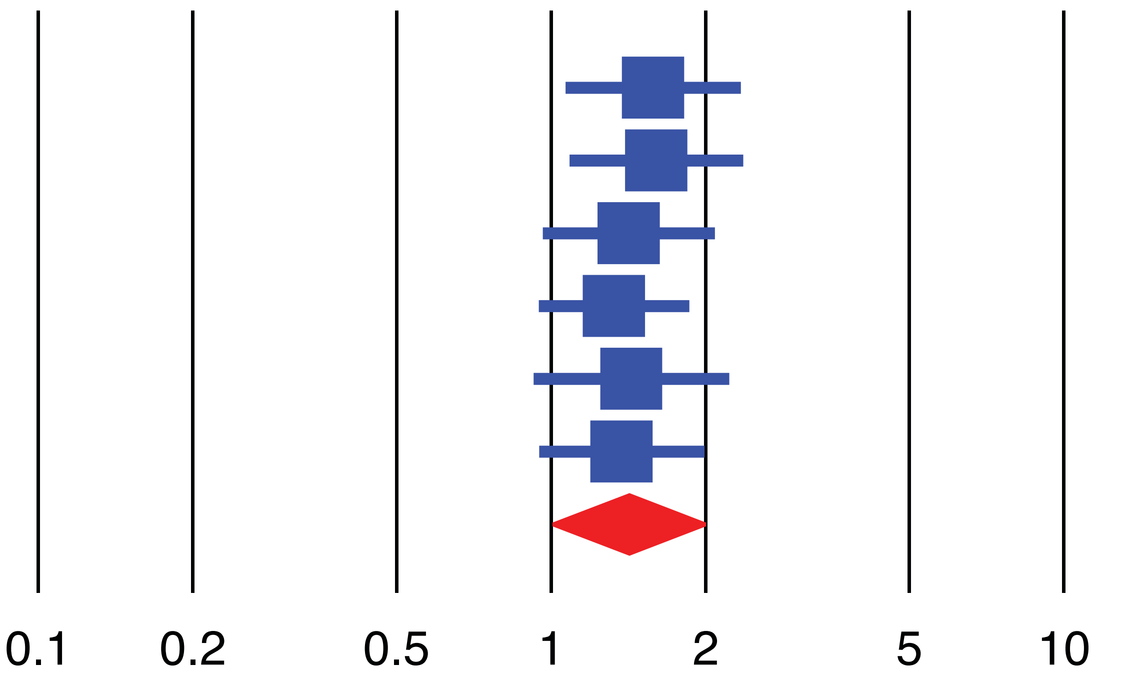

C. Recurrent MI

Study name	Point	Lower limit	Upper limit	Z-Value	p-Value
Marrow 2008	1.340	0.870	2.063	1.328	0.184
Eggers 2009	1.284	1.009	1.634	2.032	0.042
Cavusoglu 2007	1.171	0.988	1.388	1.819	0.069
Kaya 2012	1.233	0.986	1.542	1.839	0.066
Scirica BM 2011	1.407	0.996	1.988	1.937	0.053
Koch 2014	1.221	0.977	1.527	1.753	0.080
	1.241	0.996	1.545	1.927	0.054

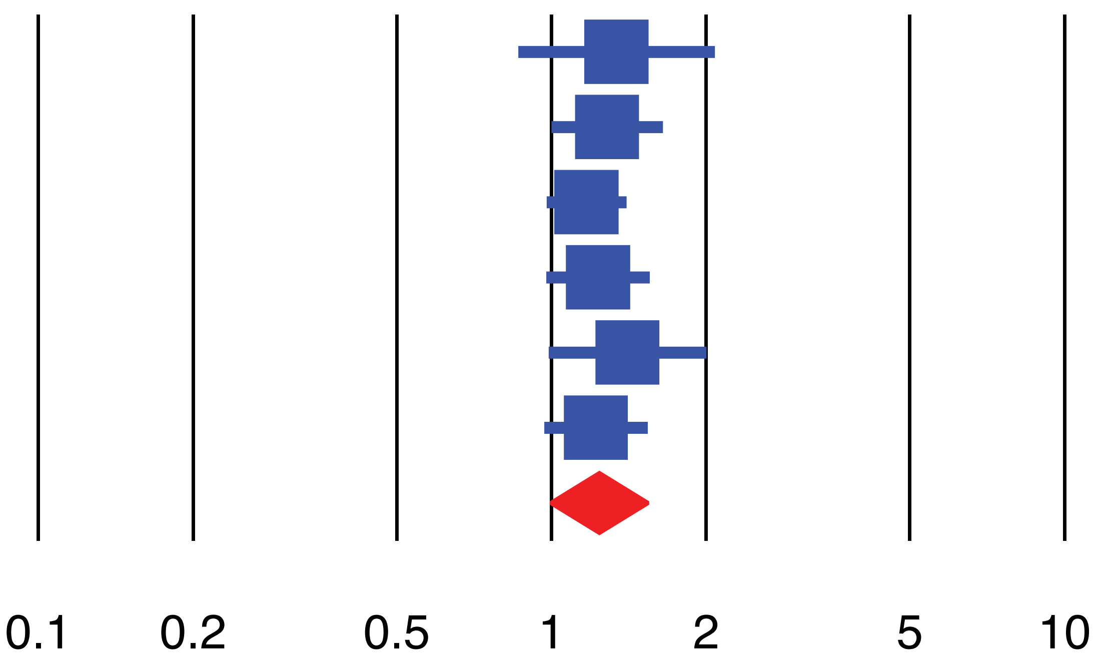

Favors low MPO **Favors high MPO**

Supplemental Figure 4.

Study name	Cumulative statistics				
	Point	Lower limit	Upper limit	Z-Value	p-Value
**A. Mortality**					
Baldus 2003	1.263	0.365	4.372	0.368	0.713
Chang 2009	4.100	0.366	45.880	1.145	0.252
Eggers 2009	3.242	1.024	10.268	2.000	0.046
Mocatta 2007	2.760	1.448	5.262	3.084	0.002
Kaya 2012	2.861	1.597	5.125	3.533	0.000
Scirica BM 2011	2.345	1.479	3.719	3.625	0.000
Rahman 2016	2.207	1.424	3.419	3.543	0.000
Koch 2014	2.253	1.552	3.270	4.271	0.000
Apple 2007	2.040	1.405	2.960	3.751	0.000
	2.040	1.405	2.960	3.751	0.000

Cumulative odds ratio (95% CI)

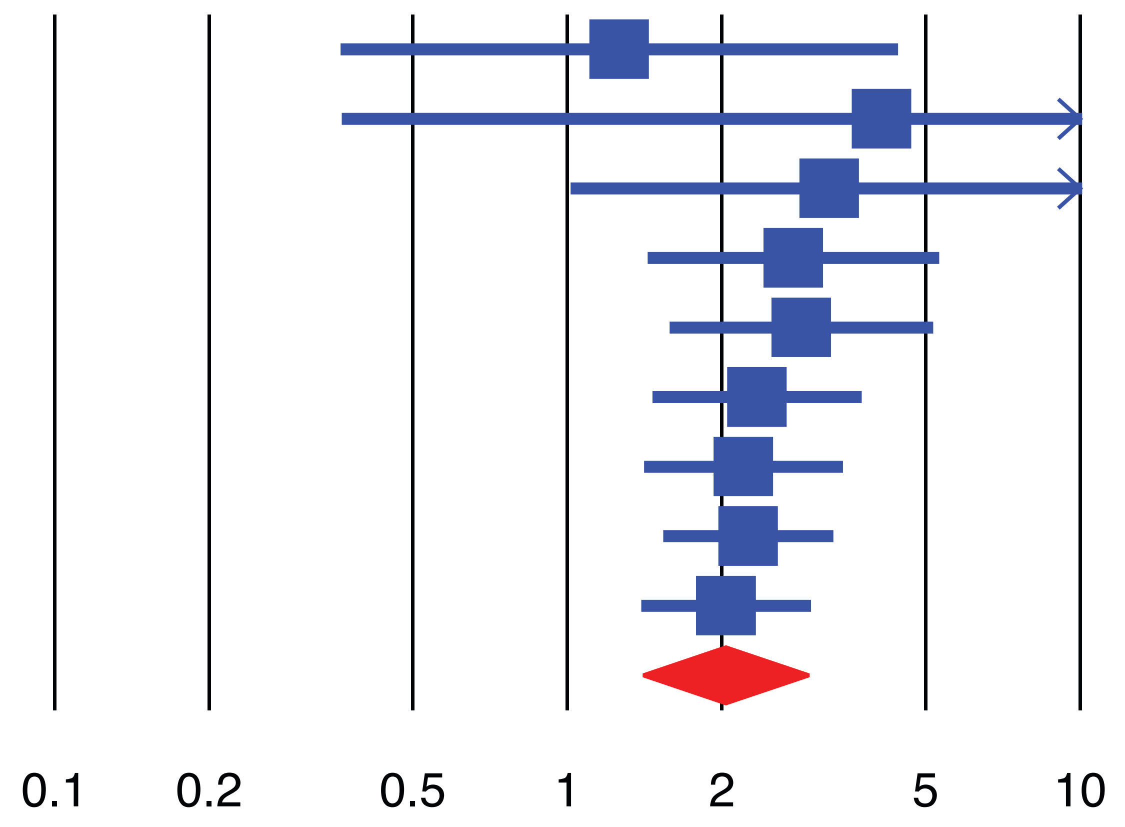

B. MACE

	Point	Lower limit	Upper limit	Z-Value	p-Value
Chang 2009	1.454	0.647	3.267	0.907	0.364
Brügger 2008	1.158	0.664	2.020	0.517	0.605
Eggers 2009	1.215	0.775	1.904	0.850	0.395
Kaya 2012	1.374	0.855	2.208	1.315	0.189
Oemrawsingh 2011	1.373	0.956	1.971	1.718	0.086
Apple 2007	1.421	1.010	1.999	2.014	0.044
	1.421	1.010	1.999	2.014	0.044

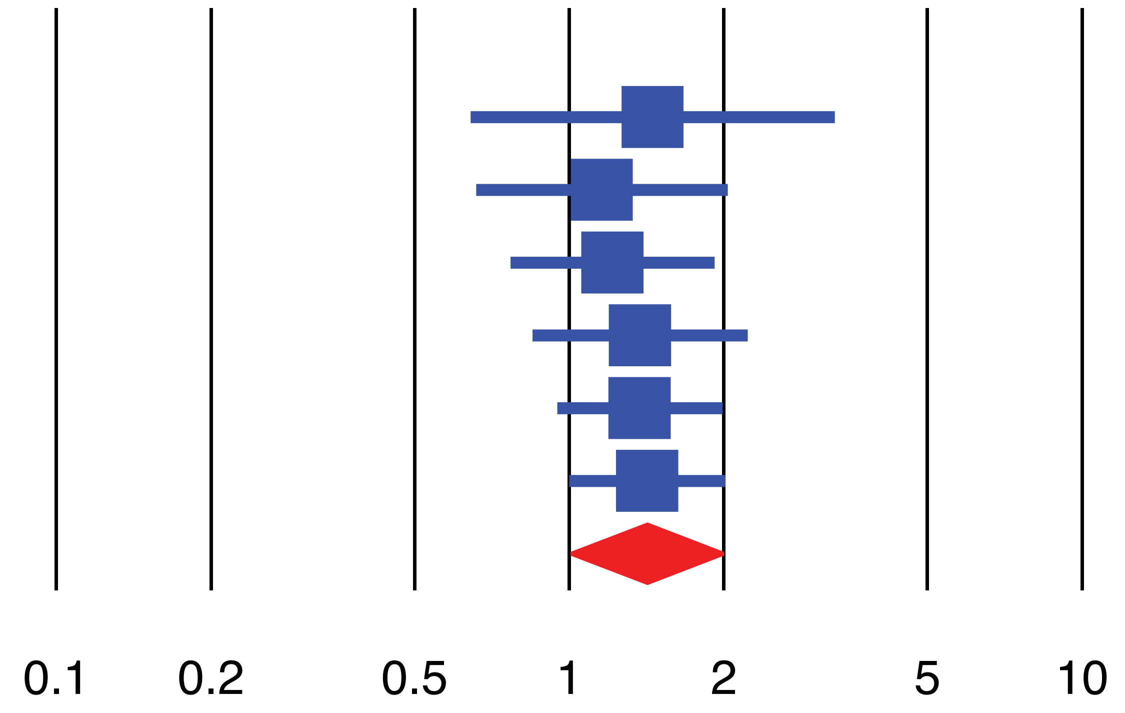

C. Recurrent MI

	Point	Lower limit	Upper limit	Z-Value	p-Value
Marrow 2008	1.280	0.965	1.696	1.715	0.086
Eggers 2009	1.231	0.940	1.612	1.510	0.131
Cavusoglu 2007	1.347	0.875	2.072	1.353	0.176
Kaya 2012	1.375	0.920	2.057	1.553	0.120
Scirica BM 2011	1.221	0.977	1.527	1.753	0.080
Koch 2014	1.241	0.996	1.545	1.927	0.054
	1.241	0.996	1.545	1.927	0.054

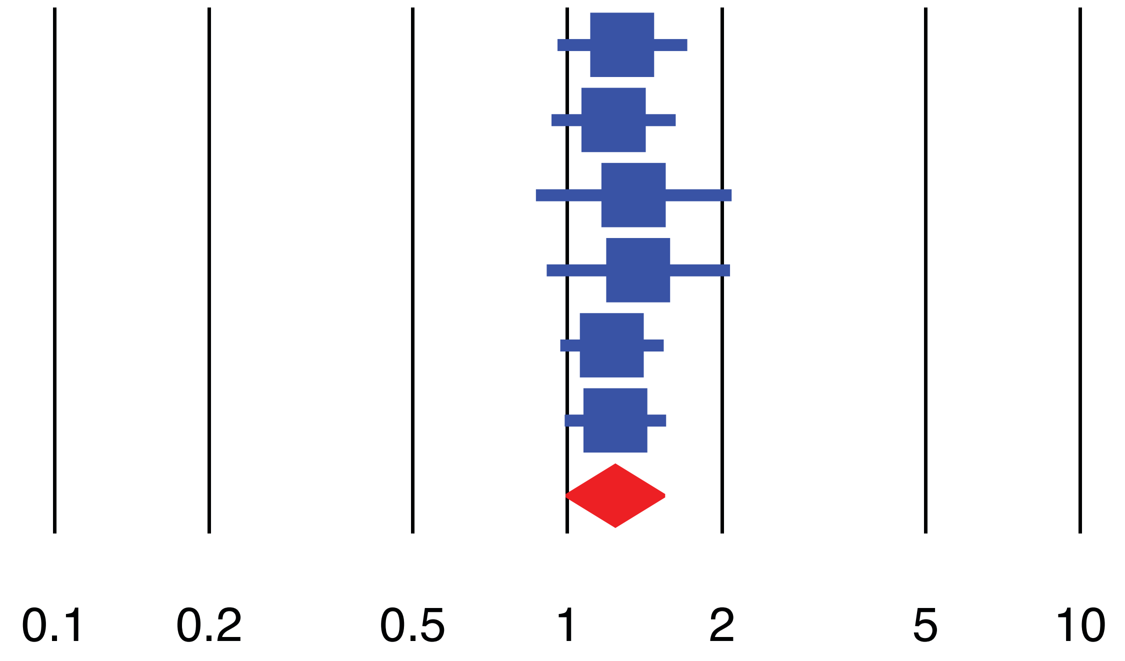

Favors low MPO   Favors high MPO

Supplemental Figure 5.

Mortality

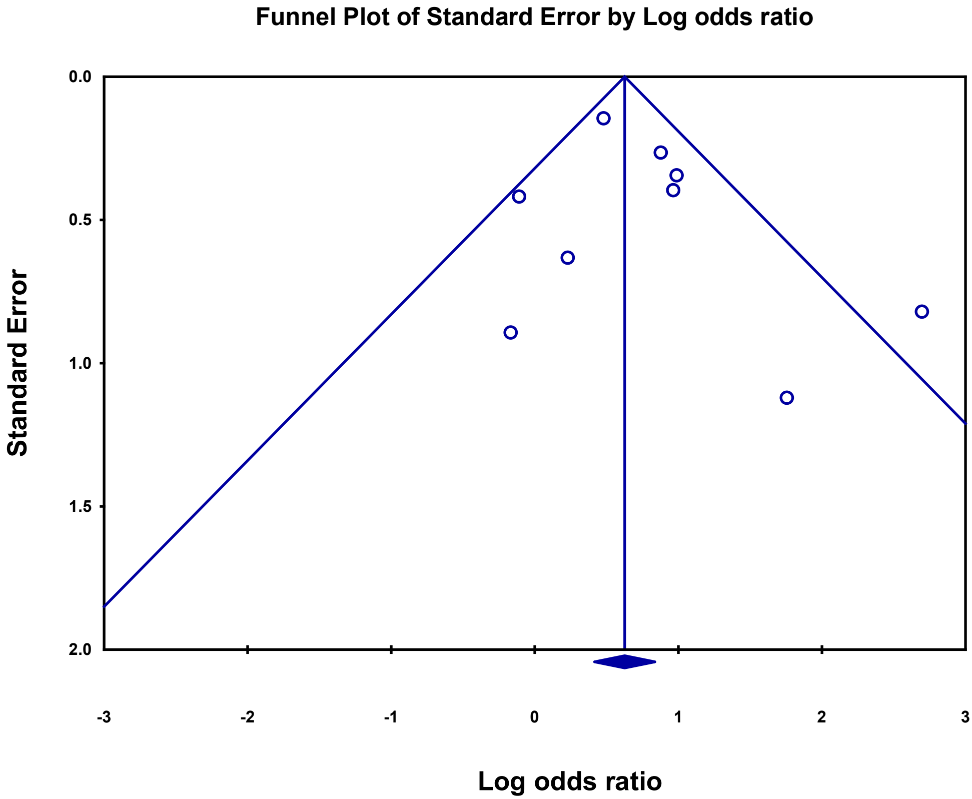

Supplemental Figure 6.

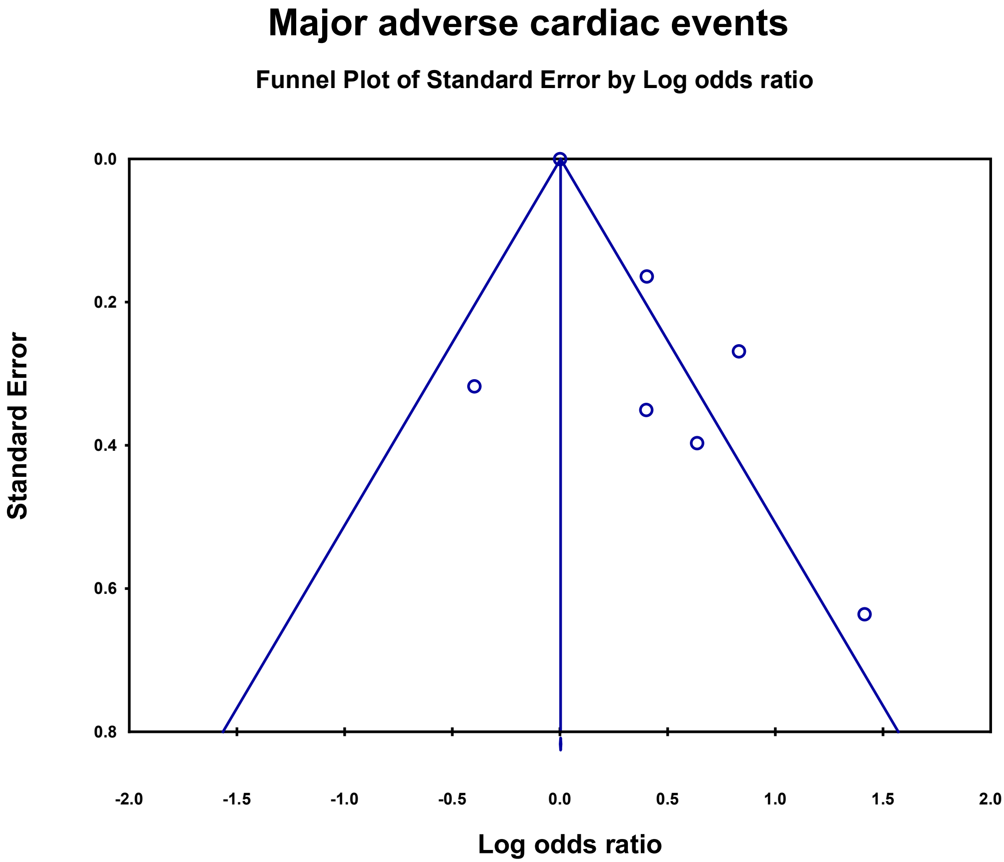

Supplemental Figure 7.

Recurrent myocardial infarction

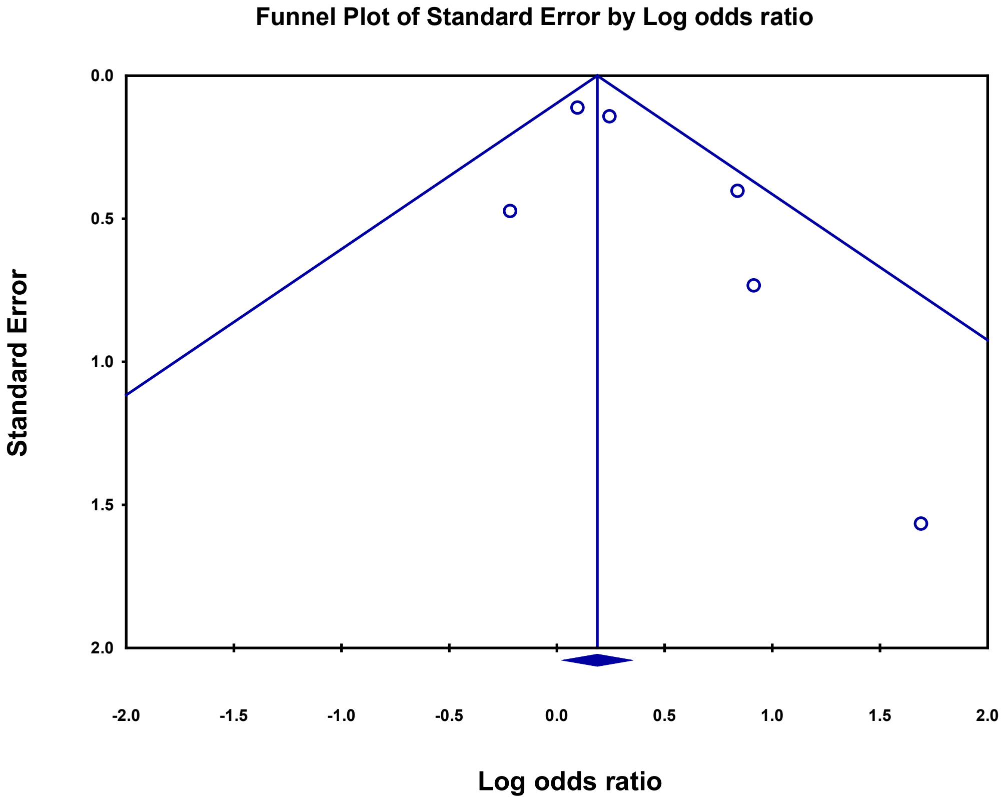

Supplemental Figure 8.
